# Predictors of coronary artery disease in cardiac arrest survivors: coronary angiography for everyone? A single-center retrospective analysis

**DOI:** 10.5935/0103-507X.20210032

**Published:** 2021

**Authors:** Joana Rigueira, Inês Aguiar-Ricardo, Pedro Carrilho-Ferreira, Miguel Nobre Menezes, Sara Pereira, Pedro S. Morais, Pedro Canas da Silva, Fausto J. Pinto

**Affiliations:** 1 Cardiology Service, Centro Hospitalar Universitário Lisboa Norte, Faculdade de Medicina, Universidade de Lisboa - Lisboa, Portugal.

**Keywords:** Cardiac arrest, Coronary artery disease, Coronary angiography, Percutaneous coronary intervention, Survival, Parada cardíaca, Doença da artéria coronária, Angiografia coronária, Intervenção coronária percutânea, Sobrevida

## Abstract

**Objective:**

To identify predictors of coronary artery disease in survivors of cardiac arrest, to define the best timing for coronary angiography and to establish the relationship between coronary artery disease and mortality.

**Methods:**

This was a single-center retrospective study including consecutive patients who underwent coronary angiography after cardiac arrest.

**Results:**

A total of 117 patients (63 ± 13 years, 77% men) were included. Most cardiac arrest incidents occurred with shockable rhythms (70.1%), and the median duration until the return of spontaneous circulation was 10 minutes. Significant coronary artery disease was found in 68.4% of patients, of whom 75% underwent percutaneous coronary intervention. ST-segment elevation (OR 6.5, 95%CI 2.2 - 19.6; p = 0.001), the presence of wall motion abnormalities (OR 22.0, 95%CI 5.7 - 84.6; p < 0.001), an left ventricular ejection fraction ≤ 40% (OR 6.2, 95%CI 1.8 - 21.8; p = 0.005) and elevated high sensitivity troponin T (OR 3.04, 95%CI 1.3 - 6.9; p = 0.008) were predictors of coronary artery disease; the latter had poor accuracy (area under the curve 0.64; p = 0.004), with an optimal cutoff of 170ng/L. Only ST-segment elevation and the presence of wall motion abnormalities were independent predictors of coronary artery disease. The duration of cardiac arrest (OR 1.015, 95%CI 1.0 - 1.05; p = 0.048) was an independent predictor of death, and shockable rhythm (OR 0.4, 95%CI 0.4 - 0.9; p = 0.031) was an independent predictor of survival. The presence of coronary artery disease and the performance of percutaneous coronary intervention had no impact on survival; it was not possible to establish the best cutoff for coronary angiography timing.

**Conclusion:**

In patients with cardiac arrest, ST-segment elevation, wall motion abnormalities, left ventricular dysfunction and elevated high sensitivity troponin T were predictive of coronary artery disease. Neither coronary artery disease nor percutaneous coronary intervention significantly impacted survival.

## Abstract (pt)

**Objetivo:**

Identificar os preditores de doença arterial coronária em sobreviventes à parada cardíaca, visando definir o melhor momento para realização de angiografia coronária e estabelecer o relacionamento entre doença arterial coronária e mortalidade.

**Métodos:**

Este foi um estudo retrospectivo em centro único, que incluiu os pacientes consecutivamente submetidos à angiografia coronária após uma parada cardíaca.

**Resultados:**

Incluímos 117 pacientes (63 ± 13 anos, 77% homens). A maioria dos incidentes de parada cardíaca ocorreu com ritmos chocáveis (70,1%), e o tempo mediano até retorno da circulação espontânea foi de 10 minutos. Identificou-se doença arterial coronária em 68,4% dos pacientes, dentre os quais 75% foram submetidos à intervenção coronária percutânea. Elevação do segmento ST (RC de 6,5; IC95% 2,2 - 19,6; p = 0,001), presença de alterações da contratilidade segmentar (RC de 22,0; IC95% 5,7 - 84,6; p < 0,001), fração de ejeção ventricular esquerda ≤ 40% (RC de 6,2; IC95% 1,8 - 21,8; p = 0,005) e níveis elevados de troponina T de alta sensibilidade (RC de 3,04; IC95% 1,3 - 6,9; p = 0,008) foram preditores de doença arterial coronária; esse último teve baixa precisão (área sob a curva de 0,64; p = 0,004), tendo o nível de 170ng/L como ponto ideal de corte. Apenas elevação do segmento ST e presença de alterações da contratilidade segmentar foram preditores independentes de doença arterial coronária. A duração da parada cardíaca (RC de 1,015; IC95% 1,0 - 1,05; p = 0,048) foi um preditor independente de óbito, e ritmo chocável (RC de 0,4; IC95% 0,4 - 0,9; p = 0,031) foi um preditor independente de sobrevivência. A presença de doença arterial coronária e a realização de intervenção coronária percutânea não tiveram impacto na sobrevivência; não foi possível estabelecer o melhor ponto de corte para o momento da angiografia coronária.

**Conclusão:**

Em pacientes com parada cardíaca, elevação do segmento ST, alterações da contratilidade segmentar, disfunção ventricular esquerda e níveis elevados de troponina T de alta sensibilidade foram preditivos de doença arterial coronária. Nem doença arterial coronária nem a intervenção coronária percutânea tiveram impacto significante na sobrevivência.

## INTRODUCTION

Sudden cardiac arrest (CA) is one of the leading causes of death in Europe and the United States.^(1,2)^ Although successful resuscitation is currently achieved in 40% - 60% of patients^(3)^ in whom advanced cardiac life support is attempted, the long-term survival rates following out-of-hospital CA remain dismal.^(4)^

The etiology of CA is diverse and includes cardiac and noncardiac causes. Evidence suggests that coronary artery disease (CAD) is a main cause of CA, with significant CAD documented by coronary angiography or autopsy in more than 70% of patients with resuscitated CA,^(3,5,6)^ but even in those patients, it is often difficult to establish whether the patient had an acute coronary event or simply bystander chronic CAD.

If acute coronary occlusion is the cause of CA, percutaneous coronary intervention (PCI) might reduce infarct size, improve hemodynamic status and reduce the recurrence of life-threatening arrhythmias and recurrent CA.^(3,7)^ Considering this evidence, international guidelines recommend coronary angiography and PCI when indicated in patients following CA with ST-segment elevation (class of recommendation I, Level of Evidence B) and when there is a high index of suspicion of ongoing infarction (class of recommendation IIa, Level of Evidence C in European guidelines and B in American guidelines).^(8,9)^

However, since patients after CA have been excluded from the main randomized trials that demonstrated the benefits of primary PCI in acute coronary syndrome (ACS) with or without ST-segment elevation, the real benefit of systematic coronary angiography in these patients is still under debate.^(3)^

Although some studies support immediate coronary angiography and possible PCI in the setting of out-of-hospital CA, as it appears to be associated with better survival at discharge,^(10,11)^ this remains controversial, and some authors highlight that routine emergency coronary angiography is probably not cost-effective or entirely risk-free.^(12,13)^ Indeed, the recently published COACT^(7)^ trial found that immediate angiography was not better than delayed angiography with respect to overall survival in these patients.

The main objective of this study was to identify independent predictors of CAD in CA survivors, with the ultimate goal of helping to determine which patients should undergo coronary angiography. The secondary objectives were to establish the best timing for coronary angiography in these patients and to determine the relationship of the presence of CAD, PCI, and mortality.

## METHODS

A retrospective single-center observational study was performed in a tertiary university hospital (Department of Cardiology, *Hospital Universitário de Santa Maria*, Lisboa, Portugal).

We included all consecutive adult patients who underwent coronary angiography in the setting of CA after the return of spontaneous circulation (ROSC) from January 2015 to July 2018.

Demographic, clinical, laboratory, electrocardiographic, echocardiographic and angiographic data were reviewed based on clinical records, which were fully electronic.

Comorbidities were acknowledged by being referenced in the patient clinical file or by laboratory results available prior to the index hospital admission (for diabetes, chronic kidney disease and dyslipidemia).

The assumed cause for CA was adjudicated by reviewing the electronic clinical records of the patient.

All electrocardiograms (ECGs) available in the electronic clinical records were reviewed by two authors to identify criteria of ST-segment elevation or equivalents according to the current recommendations.^(8)^ Other signs of ischemia (i.e., *de novo* ST-segment depression and T-wave inversion) were also recorded.

Left ventricular (LV) systolic dysfunction, based on the LV ejection fraction (LVEF), was classified according to the current European recommendations.^(14)^

Significant CAD was defined by a lesion resulting in a lumen reduction of at least 50% in the left main artery and of at least 70% in the remaining vessels. The first available laboratory results after ROSC were considered.

### Statistical analysis

The statistical analysis was performed using IBM Statistical Package for Social Disease (SPSS, Chicago, IL, United States), version 23. Categorical variables are reported in the absolute number and percentage, and continuous variables are reported as the mean and standard deviation or median and interquartile range (IQR), according to the normality of the distribution, which was tested with the Shapiro-Wilk test. Factors associated with CAD were identified by the chi-square test or Fisher’s exact test for categorical variables and Student’s t test or a nonparametric test for continuous variables with normal and nonnormal distribution, respectively. Independent predictors of CAD were identified by logistic regression analysis. Independent predictors of death were identified with Cox regression analysis. Receiver operating characteristic (ROC) curve analysis was used to determine the best cutoff of troponin level to predict CAD and the best timing for coronary angiography. A p-value below 0.05 was considered significant.

All data were analyzed anonymously. Informed Consent was waived because no change was applied to the patients’ management. The present study conformed to the ethical guidelines of the Declaration of Helsinki.

## RESULTS

The patient demographics are summarized in table 1. One hundred seventeen patients (n=117) were included, mostly male (77%, n = 90), with a mean age of 63 ± 13 years (range 30 - 97).

**Table 1:** Demographic and clinical data

Characteristic	Total (N = 117)	Significant CAD (N = 80)	No significant CAD (N = 37)	p value*
Age (years)	63 ± 13	63.7 ± 12.4	61.9 ± 13.6	0.47
Males	90 (77)	60 (75)	30 (81.1)	
Comorbidities				
Obesity	26 (25.5)	15 (21.1)	11 (35.5)	0.12
Arterial hypertension	72 (63.2)	54 (69.2)	18 (50.0)	0.048
Dyslipidemia	48 (42.5)	35 (45.5)	13 (36.1)	0.35
Diabetes	31 (27.4)	21 (26.9)	10 (28.6)	0.86
Smoking	36 (32.4)	28 (36.4)	8 (23.5)	0.18
Chronic kidney disease - Stage V	6 (5.2)	2 (2.5)	4 (11.1)	0.055
Coronary artery disease	41 (36.6)	29 (37.7)	12 (34.3)	0.73
Cerebrovascular disease	14 (12.8)	12 (15.8)	2 (6.1)	0.12

CAD - coronary artery disease.

* p indicates the difference between patients with and those without significant coronary artery disease. Results expressed as mean ± standard deviation or n (%).

Cardiovascular risk factors were common: 63% of the patients had arterial hypertension, 43% dyslipidemia, 27% diabetes and 32% a history of smoking. Thirty-seven patients had a previous history of CAD, and 13% had cerebrovascular disease.

In 24.8% of the patients, there was no information regarding the presumed cause of CA before coronary angiography. In the remainder, based on clinical judgment, type 1 myocardial infarction was the presumed cause in 79.5%, mainly with ST-segment elevation (60%). The presumed causes of CA are detailed in table 2.

**Table 2:** Presumed cardiac arrest cause

	n (%)
Type 1 myocardial infarction	70 (59.8)
STEMI	42 (35.9)
NSTEMI	28 (23.9)
Type 4A myocardial infarction - associated with PCI	2 (1.7)
Type 4A myocardial infarction - stent thrombosis	1 (0.9)
Type 5 myocardial infarction - associated with CABG	2 (1.7)
Cardiogenic shock	2 (1.7)
Other causes	11 9.5)
Unknown	29 (24.8)

STEMI - ST-segment elevation myocardial infarction; NSTEMI - non-ST segment elevation myocardial infarction; PCI - percutaneous coronary intervention; CABG - coronary artery bypass graft.

Most cases (58.2%) were out-of-hospital CA, which usually occurred in shockable rhythm (70.1%). The median time until ROSC was 10 minutes (IQR = 4 - 20). Seventy-six percent of the patients underwent coronary angiography in the first 24 hours after ROSC and 38% in the first 2 hours, with a median time of ROSC to coronary angiography of 3 hours. Table 3 summarizes the characteristics of CA.

**Table 3:** Characteristics of cardiorespiratory arrest

	Total (N = 117)	Significant CAD (N = 80)	No significant CAD (N = 37)	p value*
Time to ROSC (minutes)	10.0 (4.0 - 20.0)	13.5 (5.25 - 20.0)	8.0 (3.0 - 16.0)	0.124
Time from CA to coronary angiography (hours)	3.0 (1.6 - 22.0)	2.5 (1.5 - 11.1)	14.0 (2.63 - 99.0)	0.01
Shockable rhythm	82 (70.1)	56 (70)	26 (70.3)	0.651
Unknown CA rhythm	19 (16.2)	14 (17.5)	5 (13.5)	0.587
Out of hospital CA	57 (58.2)	37 (57.8)	20 (58.8)	0.923

CAD - coronary artery disease; ROSC - return of spontaneous circulation; CA - cardiorespiratory arrest.

* p indicates the difference between patients with and those without significant coronary artery disease. Results expressed as the median and interquartile range (Q1 - Q3) or n (%).

In coronary angiography, significant CAD was found in 68.4% (n = 80) of the patients, mainly involving the left anterior descending artery (44%; n = 52), and 14% (n = 16) had left main artery involvement. Considering patients with significant CAD, 75.0% (n = 60) underwent PCI, 12.5% (n = 10) were considered for surgical intervention, and the remaining 12.5% (n = 10) did not undergo revascularization. Among these, four patients were considered to have chronic CAD, since all of these patients had previously known lesions or occluded bypass grafts; three patients had disease in vessels of small diameter only; and the remaining three patients had chronic total occlusions. Considering patients without significant CAD, eight underwent left ventriculography, of whom 71% (n = 5) had wall motion abnormalities (WMAs); one patient had WMAs compatible with Takotsubo syndrome, and 50% (n = 4) had a severely reduced LVEF.

The results of the post-CA ECG were available in 86 patients. Of these, most did not have ST-segment elevation (54.7%, n = 47), and only 12.3% (n = 10) had left bundle branch block. However, only 7% of the patients (n = 6) had an unremarkable ECG. In the subgroup of patients without significant CAD, 17.9% (n = 5) had ST-segment elevation, 44.4% (n = 12) had other electrocardiographic signs suggestive of myocardial ischemia and 13.8% (n = 4) had an unremarkable ECG. Of the 79 patients who had available immediate post-ROSC echocardiogram results, 51% (n = 40) had WMAs, and 52% (n = 41) had at least moderate LV systolic dysfunction (LVEF ≤ 40%). In the subgroup without significant CAD, 10.7% (n = 3) had WMAs, and 37.9% (n = 11) had moderate LV systolic dysfunction.

Considering the laboratory results, only 7% of the patients (n = 7) had troponin in the normal range. In the subgroup without significant CAD, only 3.0% (n = 1) had troponin levels in the normal range. Table 4 details the results of the exams performed after ROSC.

**Table 4:** Results of complementary exams after the return of spontaneous circulation

	Total (N = 117)	Significant CAD (N = 80)	No significant CAD (N = 37)	p value*
Coronary angiography				
CAD location				
Left main	16 (13.7)			
Left anterior descending artery	52 (44.4)			
Left circumflex artery	40 (34.2)			
Right coronary artery	45 (38.5)			
Number of vessels with significant CAD				
1	38 (32.5)			
2	25 (21.4)			
3	17 (14.5)			
Transthoracic echocardiography (N = 79)				
LVEF				0.022
> 50%	34 (42.5)	16 (31.4)	18 (62.1)	
41 - 50%	5 (6.3)	5 (9.8)	0 (0.0)	
31 - 40%	21 (26.3)	17 (33.3)	4 (13.8)	
≤ 30%	20 (25)	13 (25.5)	7 (24.1)	
Presence of wmas	40 (50.6)	37 (72.5)	3 (10.7)	< 0.001
Pericardial effusion	6 (7.6)	6 (11.8)	0 (0.0)	0.84
ECG (n = 86)				
Unremarkable	6 (6.9)	2 (3.4)	4 (13.8)	0.586
Rhythm				
Sinus rhythm	63 (73.3)	44 (77.2)	19 (65.5)	
Atrial fibrillation/flutter	9 (10.5)	4 (7.0)	5 (17.2)	
Ventricular tachycardia	3 (3.5)	2 (3.5)	1 (3.4)	
Atrioventricular block	7 (8.1)	5 (8.8)	2 (6.9)	
Other rhythm	4 (4.7)	2 (3.5)	2 (6.9)	
Interventricular conduction delay				0.954
LBBB	10 (12.3)	6 (11.1)	4 (14.8)	
RBBB	14 (17.3)	9 (16.7)	5 (18.5)	
Nonspecific	9 (11.1)	6 (11.1)	3 (11.1)	
ST-segment elevation	39 (45.3)	34 (58.6)	5 (17.9)	< 0.001
Other signs of ischemia	47 (40.1)	20 (38.5)	12 (44.4)	0.607
Laboratory results (N = 99)				
Increased troponin levels	92 (92.9)	60 (90.9)	32 (97.0)	0.67
TnI (normal range < 0.04ng/mL)	0.82 (0.17 - 4.19)	1.1(0.15 - 4.68)	0.52(0.18 - 2.66)	0.586
hs-TnT (normal range < 14pg/mL)	169.5 (45.5 - 771.8)	282.5 (60.3 - 969.0)	96 (32.0 - 549.5)	0.048
White blood cells count (X109/L)	13.46 (9.9 - 17.31)	12.77 (10.15 - 16.0)	15.13(9.48 - 18.42)	0.58
Neutrophils (X109/L)	9.74(6.56 - 13.88)	9.59 (5.66 - 13.88)	10.89(7.31 - 14.8)	0.481
C-reactive protein (mg/dL)	0.3 (0.11 - 1.66)	0.3 (0.12 - 1.07)	0.35 (0.1 - 3.17)	0.708

CAD - coronary artery disease; LVEF - left ventricular ejection fraction; WMAs - wall motion abnormalities; ECG - electrocardiogram; LBBB - left bundle branch block; RBBB - right bundle branch block; TnI - troponin I; hs-TnT - high sensitivity troponin T.

* p indicates the difference between patients with and those without significant coronary artery disease. Results expressed as n (%) or the median and interquartile range (Q1 - Q3).

During follow-up, 52% of the patients died (n = 61); 14.5% (n = 17) died in the first 24 hours after CA, 27.4% (n = 32) died between 24 hours and 30 days, and 10.3% (n = 12) died after 30 days. The mean follow-up was 24.8 ± 11.9 months, and the mean time until death was 3.6 ± 9.1 months.

### Predictors of coronary artery disease

Patients with significant CAD had a numerically higher troponin levels (median 1.1 ng/mL, IQR = 0.18 - 2.66 *versus* 0.52ng/mL, IQR = 0.15 - 4.68; p = 0.586, for the 4^th^ generation troponin I - TnI - assay and 283pg/mL, IQR = 60.3 - 969 *versus* 96pg/mL, IQR = 32 - 549.5), p = 0.048 for high-sensitivity troponin T - hs-TnT). The ROC analysis showed that hs-TnT had a significant but poor accuracy in predicting CAD (AUC = 0.64, p = 0.04) with a best cutoff of 170pg/mL (sensitivity = 60.4% and specificity = 69.2%). A hs-TnT value above 170pg/mL was associated with the presence of significant CAD (76% of patients with hsTnT > 170pg/mL had significant CAD *versus* 51% of patients with hs-TnT ≤ 170pg/mL, p = 0.007). None of the other laboratory values assessed was associated with significant CAD.

ST-segment elevation was also associated with CAD (88% of patients with ST-segment elevation had significant CAD *versus* 51% of patients without ST-segment elevation; p < 0.001), as were the presence of WMAs in echocardiograms (93% of patients with WMAs had significant CAD *versus* 36% of patients without; p < 0.001) and an LVEF ≤ 40% (85% of patients with an LVEF ≤ 40% had significant CAD *versus* 47% of patients with an LVEF > 40%; p = 0.003). None of the other ECG or echocardiogram characteristics were associated with significant CAD.

Considering the comorbidities, only arterial hypertension was found to be marginally associated with significant CAD (75% of patients with hypertension had significant CAD *versus* 57% of patients without hypertension, p = 0.048).

In the univariate logistic regression analysis, the following characteristics were predictors of significant CAD (Table 5): the presence of WMAs (odds ratio - OR 22, 95% confidence interval - 95%CI 5.7 - 84.6, p < 0.001), ST-segment elevation in the post-ROSC ECG (OR 6.5, 95%CI 2.2- 19.6, p = 0.001), an LVEF ≤ 40% (OR 6.2, 95%CI 1.8 - 21.8, p = 0.005), and hs-TnT ≥ 170pg/mL (OR 3.03, 95%CI 1.3 - 6.9, p = 0.008). In the multivariate analysis, the presence of WMAs and ST-segment elevation were independent predictors of significant CAD (OR 25.5, 95%CI 4.8 - 135.4; p < 0.001, and OR 5.076, 95%CI 1.03 - 25.0; p = 0.046, respectively).

**Table 5:** Predictors of significant coronary artery disease

	Univariate analysis						
	p value	OR	95%CI	PPV %	NPV %	Sensitivity %	Specificity %
Presence of WMAs	< 0.001	22.0	5.7 - 84.6	92.5	64.1	72.6	82.3
ST-segment elevation	0.001	6.5	2.2 - 19.6	87	49	58.6	82.1
LVEF ≤ 40%	0.005	6.2	1.8 - 21.8	85.0	53.0	57.9	81.8
hs-TnT ≥170pg/mL	0.008	3.04	1.3 - 6.9	76	49	76.3	48.7

OR - odds ratio; 95%CI - 95% confidence interval; PPV - positive predictive value; NPV- negative predictive value; WMAs - wall motion abnormalities; LVEF - left ventricle ejection fraction; hs-TnT- high sensitivity troponin T.

In the subgroup of patients without ST-segment elevation, 24 (51.1%) had significant CAD, and 54.2% (n = 13) underwent primary PCI. In this subgroup, the presence of WMAs and an LVEF ≤ 40% were also associated with significant CAD: 88% of patients with WMAs had significant CAD *versus* 25% of patients without WMAs (p < 0.001), and 69% of patients with an LVEF ≤ 40% had significant CAD *versus* 21% of patients with an LVEF > 40% (p = 0.017). Both parameters were predictors of CAD (OR 22.5, 95%CI 3.9 - 128.3; p < 0.001; and OR 8.3, 95%CI 1.45 - 46.9; p = 0.017, respectively).

### The impact of coronary artery disease on mortality

Among patients with significant CAD, 56.3% (n = 45) died, and 43.8% (n = 35) survived. The presence of significant CAD was not associated with mortality (p = 0.19). Additionally, in the subgroup of patients with significant CAD who underwent PCI, 53.3% died (n = 32), and 46.7% (n = 28) survived. Percutaneous coronary intervention in patients with significant CAD was not associated with reduced mortality (p = 0.79). Additionally, there was no significant increase in mean survival time in patients with *versus* those without significant CAD (3.0 ± 9.7 *versus* 4.7 ± 8.2 months, p = 0.703).

Patients with indications for surgical revascularization had higher 30-day and overall mortality (p = 0.016 and p = 0.012, respectively).

There was no significant difference in the time from ROSC to coronary angiography between patients who survived and those who died (3.0 hours, IQR = 1.5-36 *versus* 2.9 hours, IQR = 1.9 - 21.5). Performing coronary angiography in the first 24 hours after ROSC *versus* more than 24 hours was not associated with reduced mortality (p = 0.134 for 30-day mortality and p = 0.67 for overall mortality), and the same was true for the 2-hour cutoff suggested in the guidelines^(8)^ (p = 0.35 for 30-day mortality and p = 0.27 for overall mortality). ROC curve analysis further strengthened these results (AUC = 0.5, p = 0.99).

The time until ROSC was associated with mortality. Those who did not survive to 30 days had a longer time until ROSC (8.0 minutes, IQR = 2.5 - 15.5 *versus* 15.0 minutes, IQR = 8.0 - 30.5; p < 0.001).

The results of the univariate and multivariate analyses are detailed in table 6. In the multivariate analysis, the time until ROSC was an independent mild predictor of overall mortality (OR 1.015, 95%CI 1.0 - 1.03; p = 0.048) and mortality in the first 24 hours after ROSC (OR 1.02, 95%CI 1.0 - 1.05; p = 0.022). Conversely, the presence of shockable rhythm was a strong independent predictor of survival (OR 0.4, 95%CI 0.2 - 0.9; p = 0.031).

**Table 6:** Predictors of death

	Univariate analysis			Multivariate analysis		
	p value	OR	95%CI	p value	OR	95%CI
Mortality - overall						
Time until ROSC (duration of CA)	0.027	1.04	1.0 - 1.1	0.048	1.015	1.0 - 1.05
CA with shockable rhythm	0.017	0.2	0.05 - 0.8	0.031	0.4	0.2 - 0.9
Indication for surgical revascularization	0.036	9.5	1.17 - 77.8			
Mortality in the first 30 days						
Time until ROSC (duration of CA)	0.006	1.05	1.0 - 1.1			
Indication for surgical revascularization	0.022	6.4	1.3 - 31.2			

OR - odds ratio; 95%CI - 95% confidence interval; ROSC - return of spontaneous circulation; CA - cardiac arrest.

## DISCUSSION

We evaluated the role of previous medical history, laboratory values, and electrocardiographic and echocardiographic data, usually available in the emergency department, to predict the presence of CAD in survivors of CA. More than 100 patients were included, and similar to previous studies, almost 70% had significant CAD.^(5,7,11,15,16)^ Our PCI rate (75%) was similar to previous reports, where rates ranged from 53% to 95%.^(5,11,12,15,17-21)^ This wide range of PCIs might be justified by the different definitions of significant CAD between studies. Importantly, our study also reported a sizeable proportion of patients considered for surgical intervention (13%), an outcome seldom reported in the literature.

Herein, we found a significant difference in time from CA to coronary angiography between patients with CAD and those without CAD (Table 3), which might be explained by selection bias, as patients with high pretest probability of CAD were probably underwent coronary angiography earlier, and patients with low pretest probability followed a different way of investigation of CA etiology. However, this time had no impact on survival.

The etiology of CA is often difficult to ascertain in an emergency setting, as medical history is often unavailable and complementary exams results are difficult to interpret.^(12)^ Therefore, the indications and best timing for coronary angiography in survivors of CA are very much a matter of debate.^(10,11)^

In our study, only direct evidence of cardiac abnormalities - ST-segment elevation, WMAs, at least moderate LV systolic dysfunction and an elevation of hs-TnT above 170pg/mL - were predictors of significant CAD.

The ECG role in the post-CA setting as a predictor of ACS is still debatable.^(3,19)^ ST-segment deviation might be caused by electrolyte abnormalities, defibrillation, ischemia-reperfusion injury and intravenous drugs administered during resuscitation,^(12,17,19)^ rendering the ECG difficult to interpret,^(22)^ with poor predictive values reported.^(5,11,17,19)^ However, some studies support our findings. Anyfantakis et al. studied 72 consecutive out-of-hospital CA survivors who underwent systematic emergency coronary angiography and found that ST-segment elevation after ROSC was independently correlated with the presence of CAD and strongly correlated with a final diagnosis of acute myocardial infarction.^(12)^ Additionally, Lee et al. found that ST-segment elevation and new-onset left bundle branch block are highly associated with acute coronary lesions.^(17)^

Emergency echocardiography in the post-CA setting may have limited value, as regional WMAs are not specific for acute myocardial ischemia and may be related to stunning caused by hypoperfusion or by resuscitation maneuvers.^(20)^ Notwithstanding, our findings suggest that emergency echocardiography might be of use in selecting patients for coronary angiography, especially if there are WMAs or an LVEF ≤ 40%.

In our study, 93% of the patients had elevated troponin levels irrespective of the presence of CAD. This finding was also reported in previous studies and reflects the very high sensitivity of the assay, suggesting that myocardial injury biomarkers are not reliable for the diagnosis of ACS in this setting because of their low specificity.^(12,17)^ We found that an elevated hs-TnT value, even with a cutoff value of 170pg/mL (almost 12 times the upper limit of normal), while predictive of CAD, has poor accuracy in CA survivors, in keeping with previous results.^(15)^ These findings render the usefulness of troponin level elevations limited from a clinical perspective.

Overall, our results support the current recommendations,^(8)^ where patients who survived to CA and present with ST-segment elevation or with high suspicion of ACS (here represented by LV systolic dysfunction, WMAs and elevated hs-TnT) should undergo coronary angiography. However, emergency doctors and interventional cardiologists must be aware of the limitations of these findings in this particular setting, which might lead to false-positive and false-negative results.

When managing these patients, one should consider that the treatment of the underlying cause of CA is paramount and that ACS is the most common cause of malignant ventricular arrhythmias leading to sudden cardiac death.^(6)^ Interestingly, we did not find an association between CA with shockable rhythm and CAD, which might have been the result of selection bias; the occurrence of ventricular arrhythmias rendered patients more likely to undergo coronary angiography. This finding is evidenced by the high prevalence of shockable rhythm in our cohort.

The results in the literature on the role of coronary angiography in this setting, performed as soon as possible after ROSC, with PCI as needed, are controversial. Several studies document the survival benefit of early coronary angiography and PCI in CA survivors.^(4,5,11,13,16,23)^ Here, as previously described,^(20,21,24)^ the only predictor of mortality was the time to ROSC, with shockable rhythm being predictive of survival. We found no association between the presence of significant CAD and PCI with survival. Our results are in keeping with those of Garot et al., who studied predictors of survival in CA complicated acute myocardial infarction in 186 patients and found that the rates of PCI success had no impact on survival.^(21)^ The authors concluded that although successful PCI could have played an important role in improving survival, their data indicated that it was not sufficient in itself to show any differences in both 30-day and 6-month mortality rates.^(21)^ Similarly, Anyfantakis et al. found that although PCI was warranted in one-third of patients, it was not associated with hospital survival.^(12)^ These findings might be the result of the small sample size of these studies, including ours, but also because in post-CA survival patients, other therapeutic interventions probably influence the prognosis.

As a result of these findings, we were unable to find a threshold for the best timing for coronary angiography in our patients, nor did the time until coronary angiography have any influence on survival. These findings are in line with the results of the COACT trial, a multicentric prospective study with 522 patients without ST-segment elevation after CA, which found that immediate angiography was not better than delayed angiography with respect to overall survival at 90 days.^(7)^

According to the literature, following successful resuscitation, only one-third of patients die because of cardiovascular causes; approximately one-third of deaths are secondary to central nervous system injury, while the remaining deaths are due to a variety of other reasons.^(4)^ Some authors even report that most patients die from neurological complications.^(13)^

Therefore, the absence of survival impact of early coronary angiography and PCI might be justified by the fact that immediate angiography leads to a delay in the implementation of interventions that might contribute to brain injury prevention, since the current practice in the center is to implement these measures only when the patient is admitted to the intensive care unit. In addition, performing coronary angiography in this setting requires the mobilization of an unstable patient, exposure to contrast agents, and a risk of vascular and bleeding complications,^(13)^ which can contribute to further mortality. Furthermore, even in patients who have genuine ischemic heart disease-related CA, some may have chronic ischemic heart disease that provides an arrhythmogenic substrate for CA. Although these patients are expected to have significant CAD, it is unclear how angiography and PCI would improve their outcomes.^(13)^

Therefore, while the identification of an acute culprit coronary lesion amenable to urgent PCI may be important for the prognosis of CA survivors, this is not the only factor involved, and careful, individual evaluation is critical. Attempts to reduce the time to ROSC and to improve post-ROSC care are probably at least as important as coronary angiography. Further randomized studies with more patients and a prospective design are essential to define the appropriate pathway for therapeutic interventions in survivors of CA - especially for selecting who should undergo coronary angiography and when.

This study has several limitations. It is a single-center retrospective study based on the information available in the electronic clinical records, and as a result, missing data are inevitable; specifically, there was a high number of patients without information regarding the post-ROSC ECG, since many of these patients are referred from other hospitals or the ECG is performed in the prehospital setting. Additionally, there was selection bias, as all patients included underwent coronary angiography based on the clinical decision of the emergency physician and the interventional cardiologist, rendering a high pretest probability, so the prevalence of significant CAD might be overrepresented. As in previous studies, the sample size was small. Another limitation was the use of two different troponin assays (TnI and hs-TnT) because we included the period of transition to a high sensitivity assay in our institution; thus, only a few patients had TnI values available. Finally, CAD was classified as significant based on the visual appreciation of the interventional cardiologist and not a core laboratory; therefore, operator bias cannot be ruled out.

## CONCLUSION

Our study suggests that in survivors of cardiac arrest, only direct evidence of cardiac abnormalities is strongly associated with coronary artery disease. ST-segment elevation in the post-return of spontaneous circulation electrocardiogram, the presence of wall motion abnormalities, an left ventricular ejection fraction ≤ 40% in transthoracic echocardiography, and an high sensitivity troponin T level above 170pg/mL were predictors of significant coronary artery disease. These results may help in the selection of patients for coronary angiography, thereby reducing unnecessary procedures, and may support current guidelines, which recommend performing coronary angiography in patients with a high index of suspicion of ongoing infarction.

However, in our population, the time until coronary angiography, the presence of significant coronary artery disease and the performance of percutaneous coronary intervention did not influence survival, and it was not possible to establish the best cutoff for coronary angiography timing. This outcome was likely the result of the small sample size and confounding factors, especially noncardiovascular issues affecting prognosis.
